# Non-muscular myosin light chain kinase triggers intermittent hypoxia-induced interleukin-6 release, endothelial dysfunction and permeability

**DOI:** 10.1038/s41598-017-13268-5

**Published:** 2017-10-20

**Authors:** Sylvain Recoquillon, Manuel Gómez-Guzmán, Marion Rodier, Camille Koffi, Mathieu Nitiéma, Frédéric Gagnadoux, M. Carmen Martínez, Ramaroson Andriantsitohaina

**Affiliations:** 1 INSERM UMR1063, Stress Oxydant et Pathologies Métaboliques, UNIV Angers, Université Bretagne Loire, Angers, France; 2 Centre Hospitalo-Universitaire d’Angers, Angers, France

## Abstract

Obstructive sleep apnea is characterized by intermittent hypoxia (IH) which alters endothelial function, induces inflammation and accelerates atherosclerosis-induced cardiovascular diseases. The non-muscular myosin light chain kinase (nmMLCK) isoform contributes to endothelial cell-cell junction opening. Deletion of nmMLCK protects mice from death in septic shock models and prevents atherosclerosis in high-fat diet-fed mice. The aim of the study was to analyze the implication of nmMLCK in IH-induced vascular inflammation. Human aortic endothelial cells were exposed to 6 hours of IH in absence or presence of nmMLCK inhibitors, ML-7 (5 µM) or PIK (150 µM). IH increased reactive oxygen species (ROS) and nitric oxide (NO) production, p65-NFκB activation and IL-6 secretion. While nmMLCK inhibition did not prevent IH-induced ROS production and p65-NFκB activation, it decreased NO production and partially prevented IL-6 secretion. IH-induced IL-6 secretion and vesicle-associated membrane protein-associated vesicles re-organization were inhibited in presence of the inhibitor of protein secretion, brefeldin A, or ML-7. IH increased monocytes transendothelial migration that was partially prevented by ML-7. Finally, IH reduced endothelium-dependent relaxation to acetylcholine of aortas from wild-type but not those taken from nmMLCK-deficient mice. These results suggest that nmMLCK participates to IH-induced endothelial dysfunction resulting from cytokines secretion and endothelial permeability.

## Introduction

Obstructive sleep apnea (OSA) is a disorder characterized by repetitive partial (hypopnea) or complete (apnea) obstructions of the upper airway during sleep inducing an intermittent hypoxia (IH). OSA has been recognized as a risk factor for the development of cardiovascular complications such as hypertension, stroke, or heart failure for example^1^. Actually, IH can alter the functions of several cells among the vasculature and more precisely the endothelial cells^2^. Several studies have shown that IH increases oxidative stress, reduces nitric oxide (NO) availability and activates inflammatory pathways in endothelial cells^2–4^. As well, it has been shown that IH could activate subunits of the nuclear factor kappa B (NFκB) transcription factors family, such as p50 and p65, responsible for the activation of inflammatory genes including interleukin 6 (IL-6) or tumor necrosis factor alpha (TNFα)^5^. This inflammatory environment is responsible for the recruitment and the transendothelial migration of certain inflammatory cells, as monocytes. Recently, it has been reported an increase of monocyte adhesion on an endothelial monolayer after their exposure to IH^6^. This effect was accentuated by the co-stimulation with the monocyte chemoattractant protein 1. The adhesion of the monocytes is the first step before the transmigration across the endothelium. Evidence has been provided that mice exposed to IH had a higher risk to developed atherosclerosis compared to control mice, highlighted by the increase in intima/media thickness as well as the infiltration of lymphocytes in the vascular wall^7^. But to our knowledge, the exact mechanisms by which IH increases atherosclerosis risks, and ultimately the associated morbidity, are not fully elucidated.

The non-muscular myosin light chain kinase (nmMLCK) is a member of MLCK family. This kinase is mostly expressed in endothelial cells, monocytes, and platelets in comparison with the muscular isoforms. nmMLCK phosphorylates the myosin light chain leading to changes in cytoskeleton architecture and retraction of the cells^8^. In endothelial cells, this retraction is associated with endothelial permeability enhancement and vascular leakage. We and others have shown that nmMLCK could participate to inflammatory processes. We provided evidence that nmMLCK is involved in lethal complications as well as in the vascular reactivity changes associated with endotoxic shock. nmMLCK is linked to lipopolysaccharide (LPS)-induced up-regulation of NFκB and increased oxidative and nitrative stresses^9^. Moreover, pharmacological inhibition of nmMLCK by its inhibitor ML-7 prevents activation of p65-NFκB pathway, illustrated by the reduction of p65 and IκB phosphorylation on the serine 536 and the serine 32, respectively^10^. Moreover, Tauseef and colleagues^11^ showed that an *in vitro* nmMLCK deficiency prevents LPS-induced p65-NFκB activation pathway. Finally, Sun and colleagues^12^ reported that nmMLCK deficiency prevents accumulation of lipid drop in aorta of mice, and therefore a decrease in vascular leakage and complications in mice fed with high-fat diet.

Taken together, these results suggest a pivotal role of nmMLCK in inflammation in different models. In this study, we hypothesize that nmMLCK participates to the inflammation associated with IH in an *in vitro* model for OSA, and we evaluate the molecular implication of nmMLCK on inflammation, endothelial dysfunction and the early stage of atherosclerosis processes associated with IH.

## Results

### Experimental protocol of IH reproduces endothelial inflammation and dysfunctions of OSA

**Figure 1:**
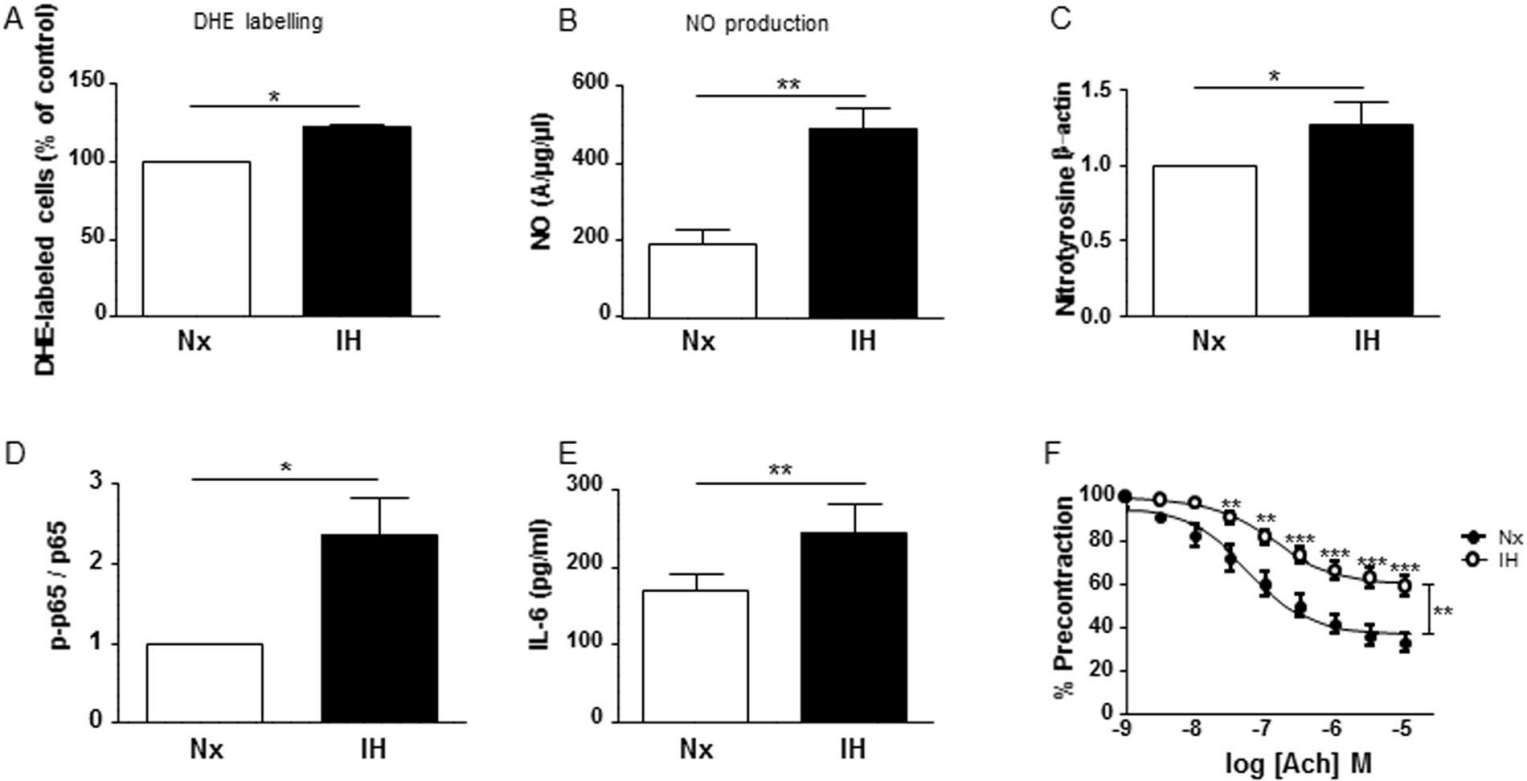
Experimental protocol of IH reproduces endothelial inflammation and dysfunctions of OSA. (**A**) Quantification of superoxide anion production in HAoECs labelled with dihydroethidium in normoxia (Nx) or 6 h of intermittent hypoxia (IH). Values represent the percentage of labelled cells (n = 5). (**B**) Quantification of the amplitude of the NO-Fe(DETC)_2_ complex signal in HAoECs exposed to Nx or IH and *in situ* NO production was determined by EPR. Values are expressed in units of amplitude per protein concentration (n = 5). (**C**) Western blot analysis of nitrotyrosine in HAoECs exposed to Nx or IH. β-actin is considered as a control for the loading amounts of proteins. Histograms show densitometric analysis of protein expression (n = 3). (**D**) Western blot analysis phosphorylation of p65 subunit of NFκB (p65) in HAoECs exposed to Nx or IH. Histograms show densitometric analysis (n = 4). (**E**) Quantification of IL-6 in the supernatant of HAoECs exposed to Nx or IH. Histograms show the concentration of IL-6 in pg/ml (n = 10). (**F**) Effect of IH exposure on concentration-response curves to acetylcholine (Ach) in aortic rings with intact endothelium pre-contracted with the thromboxane A2 analogue (U46619), from nmMLCK +/+ mice (n = 7). *P < 0.05, **P < 0.01, ***P < 0.001.

First, we wanted to explore the effects of the experimental protocol of IH exposure on human aortic endothelial cells (HAoECs). IH increased ROS and NO production, leading to an increase of protein nitration compared to control HAoECs (Fig. 1A–C). Also, IH increased the phosphorylation at the serine 536 site of p65-NFκB, a sign of p65-NFκB activation (Fig. 1D). Moreover, IH enhanced the secretion of IL-6 in the supernatant of HAoECs compared to cells exposed to normoxia (Fig. 1E). Finally, aorta exposed to the same protocol of IH displayed reduced endothelium-dependent relaxation in response to acetylcholine compared to that of aorta exposed to normoxia (Fig. 1F). Altogether, these results suggest that experimental IH exposure induces both pro-inflammatory response and endothelial dysfunction, and therefore validate this protocol as an experimental model of IH identical to vascular alterations observed in OSA.

### nmMLCK activity regulates the IH-induced increase of NO production

**Figure 2:**
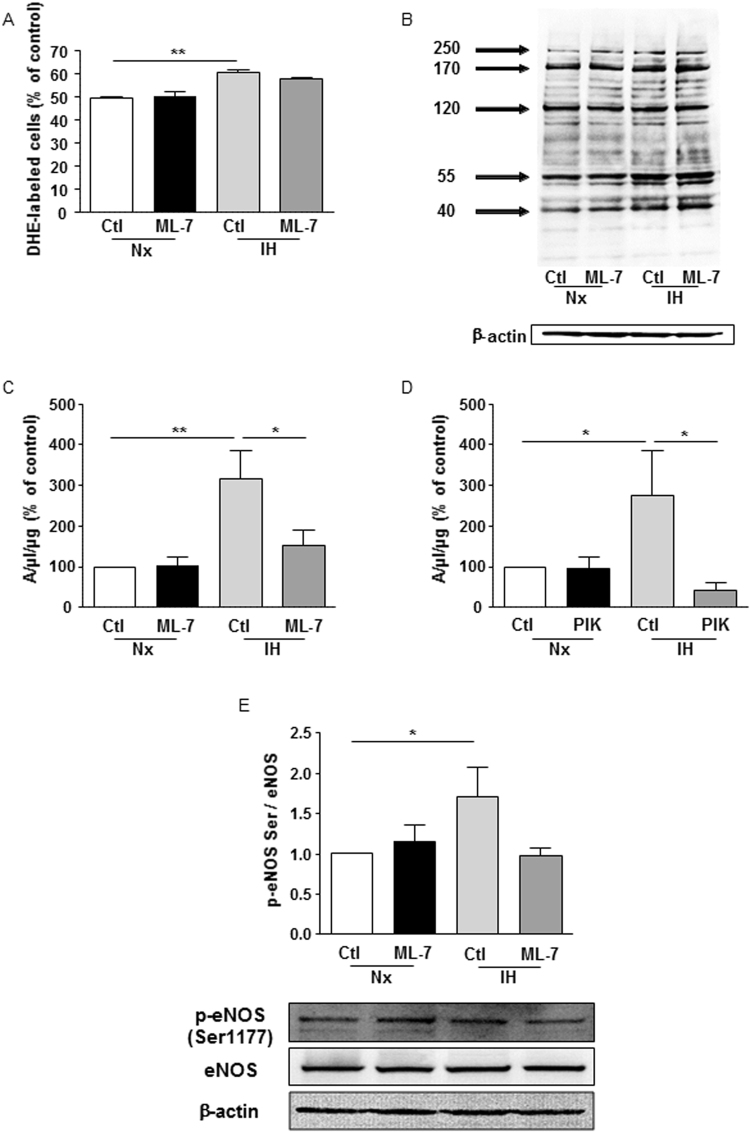
nmMLCK activity regulates the IH-induced increase of NO production. (**A**) Quantification of superoxide anion production in HAoECs labelled with dihydroethidium in normoxia (Nx) or 6 h of intermittent hypoxia (IH) in absence or presence of ML-7 (5 µM). Values represent the percentage of labelled cells (n = 5). (**B**) Western blot analysis of nitrotyrosine in HAoECs exposed to Nx or IH in absence or presence of ML-7 (5 µM). β-actin is considered as a control for the loading amounts of proteins. Histograms show densitometric analysis of protein expression (n = 3). Quantification of the amplitude of the NO-Fe(DETC)_2_ complex signal in HAoECs exposed to Nx or IH in absence or presence of ML-7 (5 µM) (**C**) or PIK (150 µM) (**D**). *In situ* NO production was determined by EPR. Values are expressed in units of amplitude per protein concentration (n = 5). (**E**) Western blot analysis of phosphorylation (Serine 1177) and expression of eNOS in HAoECs exposed to Nx or IH in absence or presence of ML-7 (5 µM). β-actin is considered as a control for the loading amounts of proteins. Histograms show densitometric analysis (n = 3). *P < 0.05, **P < 0.01.

To analyze the implication of nmMLCK, two different inhibitors acting through two different mechanisms have been used. ML-7, an inhibitor of nmMLCK at the ATP site, and the MLCK peptide inhibitor 18 (PIK), that binds to the catalytic domain of the kinase. ML-7 did not affect either the increase in ROS, or the protein nitration resulting from ROS and NO reaction induced by IH (Fig. 2A,B). Interestingly, nmMLCK inhibition either by ML-7 or PIK prevented the IH-induced increase of NO (Fig. 2C), resulting from the activation of eNOS as shown by the reduced phosphorylation of eNOS at its activator site, the serine 1177 (Fig. 2D). We were not able to detect the expression of the iNOS isoform under the experimental conditions used (data not shown). Altogether, these results suggest that nmMLCK activity regulates the production of NO by modulating the active phosphorylated form of eNOS.

### nmMLCK regulates IH-induced IL-6 release downstream of p65-NFκB activation

**Figure 3:**
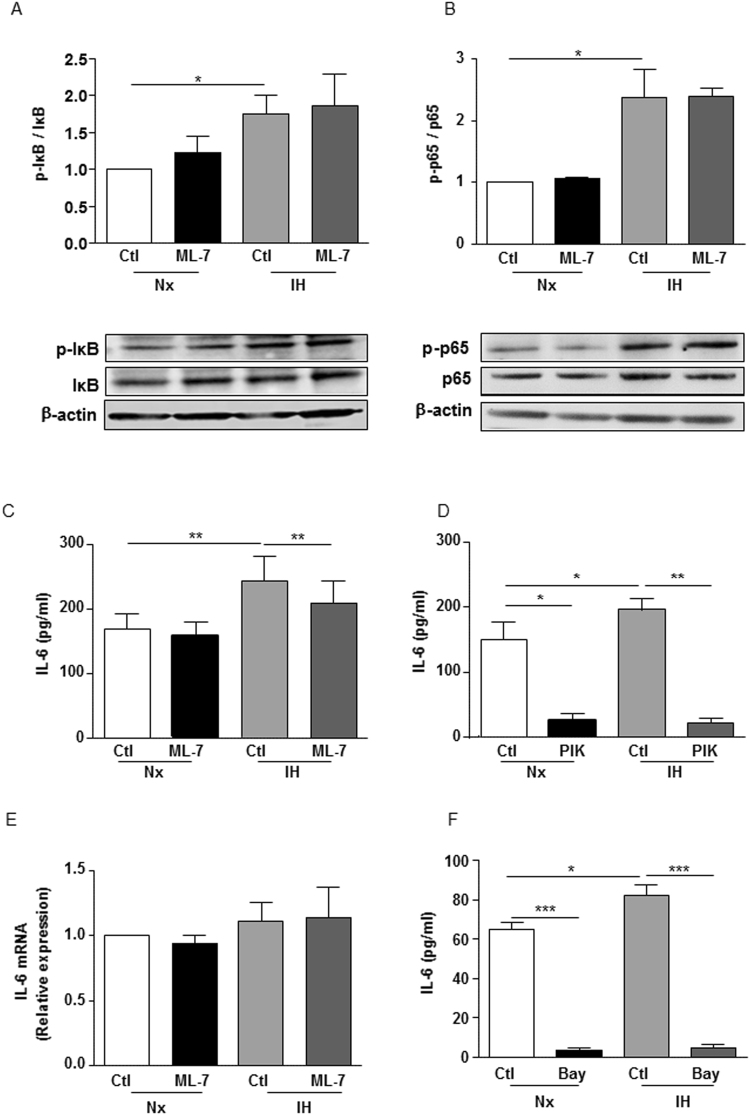
nmMLCK regulates IH-induced IL-6 release downstream of p65-NFκB activation. Western blot analysis of phosphorylation and expression of (**A**) IκBα and (**B**) p65 subunit of NFκB (p65) in HAoECs exposed to Nx or IH in the absence or presence of ML-7 (5 μM). β-Actin expression for each sample is considered as a control for the loading amount of proteins. Histograms show densitometric analysis (n = 4). Quantification of IL-6 in the supernatant of HAoECs exposed to Nx or IH in absence or presence of ML-7 (5 µM) (**C**) and PIK (150 µM) (**D**). Histograms show the concentration of IL-6 in pg/ml (n = 10). (**E**) Analysis of IL-6 mRNA by RT-PCR in HAoECs exposed to Nx or IH in absence or presence of ML-7 (5 µM). Histograms show relative expression (n = 5). (**F**) Quantification of IL-6 in the supernatant of HAoECs exposed to Nx or IH in absence or presence of an inhibitor of p65-NFκB, Bay 11–7082 (15 µM). Histograms show the concentration of IL-6 in pg/ml (n = 5). *P < 0.05, **P < 0.01, ***P < 0.001.

IH activates the transcription factor p65-NFκB. This activation requires the ubiquitin-dependent proteasome degradation of IκB, a physiological repressor of p65-NFκB, depending on the phosphorylation rate of IκB at the residue serine 32^13^. IH exposure enhanced phosphorylation of IκB (Fig. 3A) and p65-NFκB (Fig. 3B) both of which were not sensitive to nmMLCK inhibition by ML-7.

Since elevated levels of IL-6 have been reported in the plasma of patients affected by OSA^14^, we wanted to ensure whether IL-6 could be the link between IH and p65-NFκB activation. As shown on Fig. 3C, IH increased the release of IL-6 by HAoECs. ML-7 did not affect basal IL-6 release under normoxia condition, but it markedly reduced the increase of IL-6 release evoked by IH (Fig. 3C). PIK inhibited IL-6 release both at basal and under hypoxic conditions, showing that nmMLCK activity regulates IL-6 release. Of note is that the increase of IL-6 release by IH did not arise from a transcriptional activation of IL-6 gene since IH did not modify the expression of IL-6 mRNA and this in absence or in presence of ML-7 (Fig. 3D).

To test whether the release of IL-6 upon IH activation requires NFκB activation, the effect of an inhibitor of p65-NFκB, Bay 11-7082, was tested^15^. Bay 11-7082 markedly inhibited the release of IL-6 both in normoxia and in IH conditions suggesting that activation of NFκB was mandatory for its release (Fig. 3E).

### nmMLCK regulates IH-induced assembly of VAMP-dependent vesicles to trigger IL-6 exocytosis

**Figure 4:**
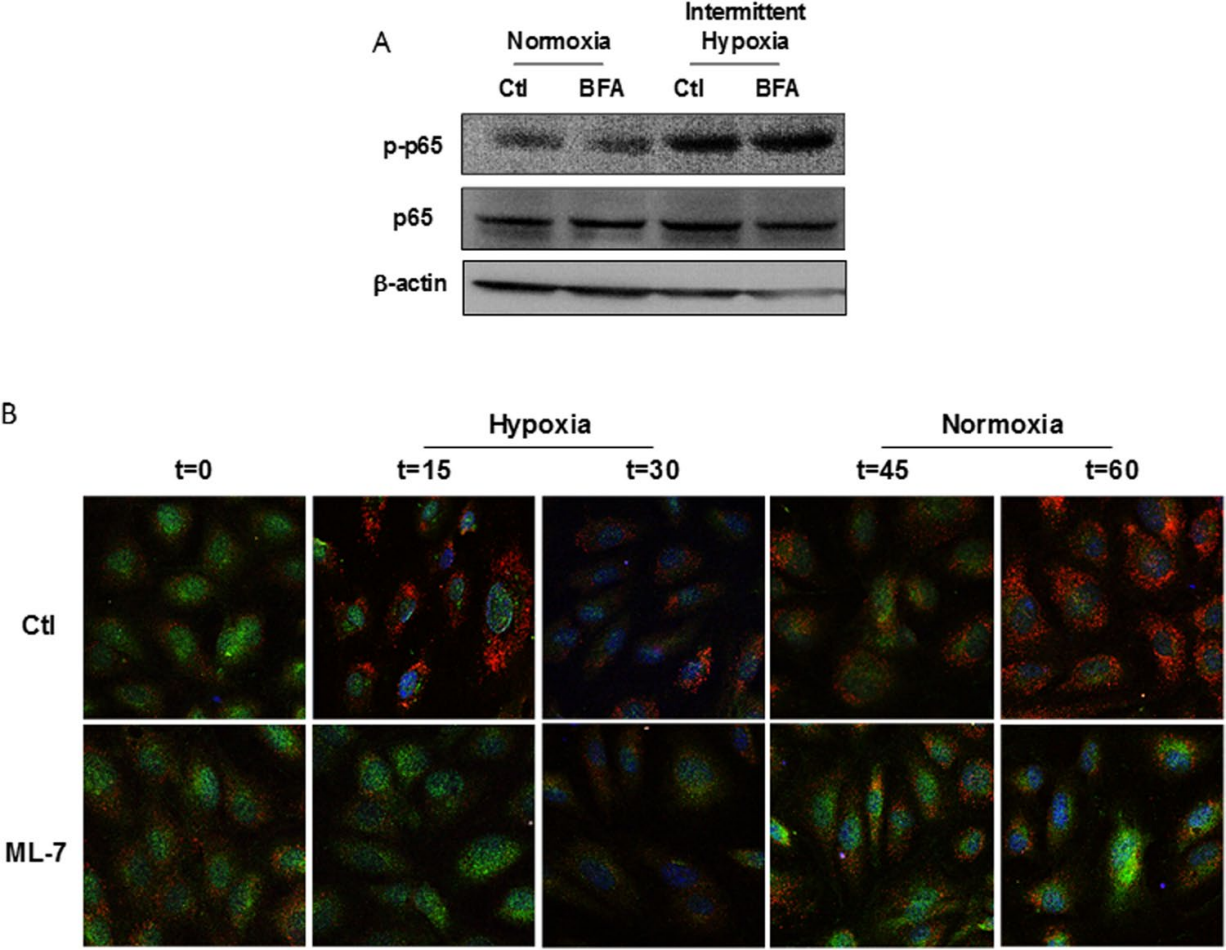
nmMLCK regulates IH-induced assembly of VAMP-dependent vesicles to trigger IL-6 exocytosis. (**A**) Western blot analysis phosphorylation of p65 subunit of NFκB (p65) in HAoECs exposed to Nx or IH in absence or presence of an inhibitor of exocytosis, brefeldin A (BFA, 10 µM) (n = 3). (**B**) Confocal microscopy analysis of HAoECs exposed to one cycle of IH in absence or presence of ML-7 (5 µM). Images were taken at 0–60 minutes of stimulation. HAoECs were labelled with an antibody directed against IL-6 (green) and VAMP (red). DAPI (blue) were used to labelled the nucleus. Images are representative of 3 independent experiments.

To investigate further the mechanism by which nmMLCK regulates IH-induced IL-6 release, we tested the effect of the exocytosis inhibitor, brefeldin A (BFA), on the secretion of IL-6. First, we wanted to ensure that BFA had no effect on normoxia and IH-induced activation of NFκB. As shown on Fig. 4B, BFA was not able to modify p65-NFκB phosphorylation both under normoxia and IH exposure reinforcing the notion that NFκB activation was upstream to the exocytosis process sensitive to BFA.

IL-6 could be stored and readily released by cells in response to multiple inflammatory stimuli to rapidly defend organism against external aggression. To evaluate the mechanism by which nmMLCK modulates IL-6 exocytosis, we localized both IL-6 and VAMP by immunofluorescence (Fig. 4B). VAMP belongs to the soluble N-ethylmaleimide-sensitive-factor attachment protein receptor (SNARE) family proteins associated with exocytosis vesicles. The experiments have been performed during one cycle of IH, at 0, 15, 30, 45 and 60 minutes, the first 30 minutes is in hypoxia and the last 30 minutes in normoxia. In the absence of ML-7, during the hypoxic phase, a decrease in IL-6 staining (green) was observed between 0 and 30 minutes. After returning to normoxic phase, IL-6 staining returned to basal values at 45 minutes and then decreased at 60 minutes. Interestingly, VAMP^+^ vesicles were arranged in the cytosolic compartment and around the plasma membrane, especially at 15 and 60 minutes. Thus, the reorganization of VAMP structures was associated with decreased IL-6 staining. In cells pre-treated with ML-7, the decrease of IL-6 was less pronounced compared to the control at 15 minutes. Moreover, during the hypoxic phase, ML-7 did not affect VAMP staining. Interestingly, during the reoxygenation phase, ML-7 reduced VAMP staining and partially prevented the decreased of IL-6 staining. Thus, ML-7 inhibited VAMP reorganization resulting in the increased storage of IL-6 within the HAoECs.

These results are in favor of the hypothesis that nmMLCK modulates IL-6 exocytosis, by a mechanism dependent on the reorganization of intracellular VAMP-carrying vesicles.

### IH increases early stages of atherosclerosis in a nmMLCK-dependent manner

**Figure 5:**
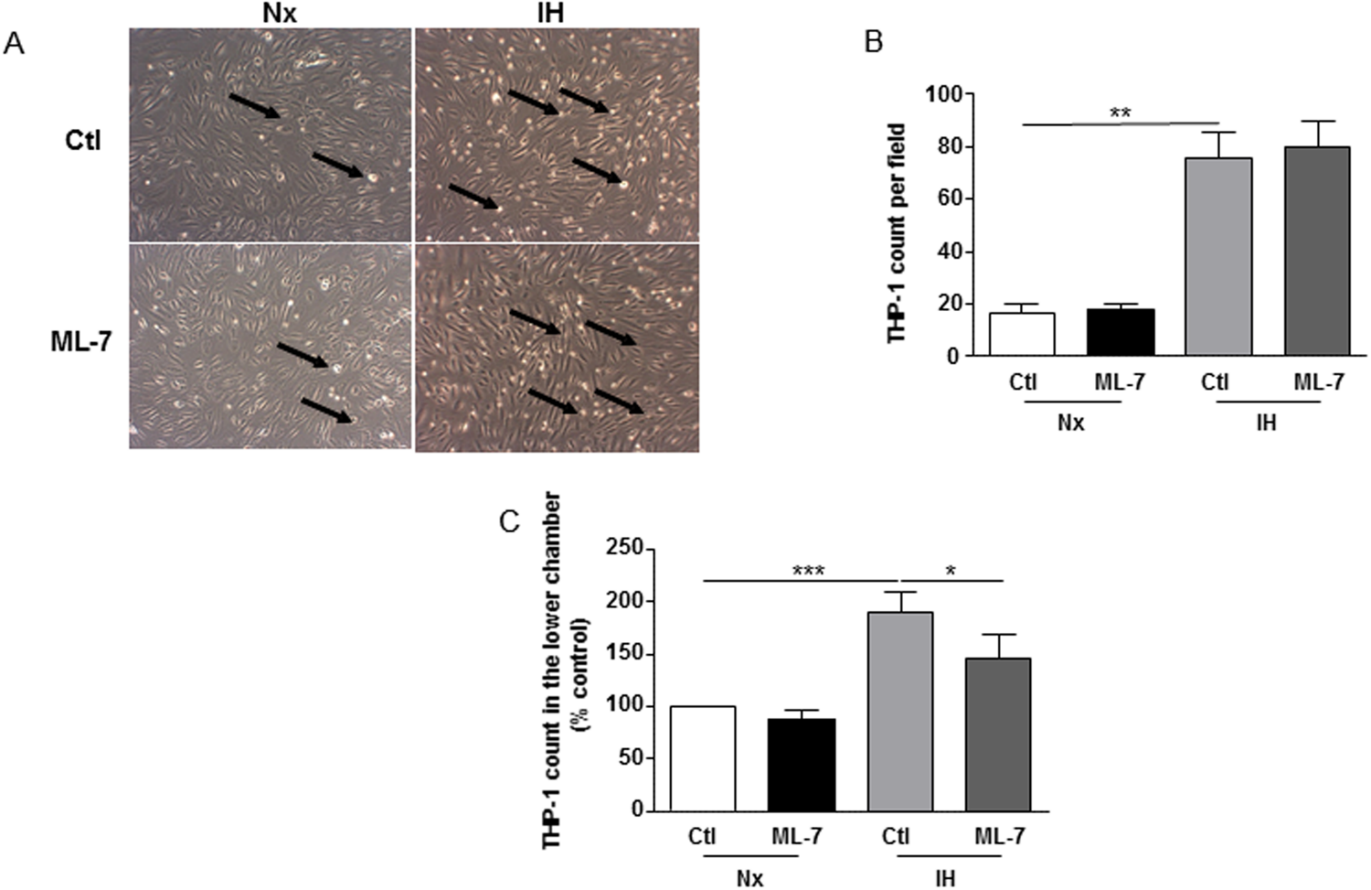
IH increases early stages of atherosclerosis in a nmMLCK-dependent manner. (**A**) Analysis of adhesion of THP-1, a monocytic cell line, on HAoECs exposed to Nx or IH in absence or presence of ML-7 (5 µM). Images are representative of 3 independent experiments. Arrows show THP-1 cells. (**B**) Quantification of the adhesion assay. THP-1 cells per field were count on 4 different images per condition (n = 3). (**C**) Analysis of transmigration of THP-1 across a layer formed by HAoECs exposed to Nx or IH in absence or presence of ML-7 (5 µM) using Transwell. Histograms represent the number of THP-1 count in the lower chamber (n = 10). *P < 0.05, **P < 0.01, ***P < 0.001.

As IH could exacerbate atherogenic process in mice (7), we wanted to explore the effect of IH on atherogenic mechanisms including adhesion of monocytes on endothelial cells as well as transendothelial migration. To assess these processes, only HAoECs were exposed to IH. For the adhesion assay, IH increased the adhesion of THP-1 on endothelial cells. ML-7 was not able to modulate the adhesion of THP-1 to endothelial cells (Fig. 5A,B). After, we evaluated the transendothelial migration of THP-1, using Transwell assay. Under the same conditions, IH increased the transmigration of THP-1 as indicated by the increase of THP-1 count in the lower chamber compared to normoxia. ML-7 alone did not modify the basal transendothelial migration but partially prevented the IH-induced increase of transendothelial migration of THP-1 (Fig. 5C). These results suggest that nmMLCK is involved in the IH-induced early stages of atherosclerotic process especially during the monocytic transendothelial migration phase.

### Deletion of nmMLCK abrogated IH-induced endothelial dysfunction

**Figure 6:**
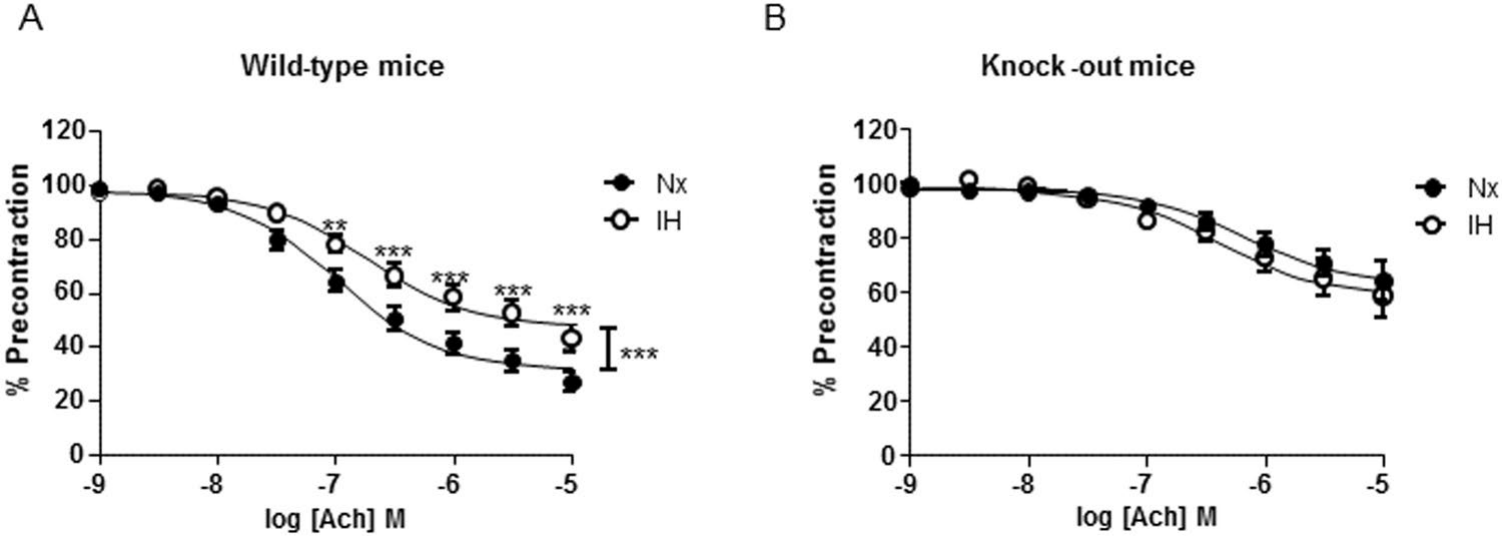
Deletion of nmMLCK abrogated IH-induced endothelial dysfunction. Effect of IH exposure on concentration-response curves to acetylcholine (Ach) in aortic rings with intact endothelium pre-contracted with the thromboxane A2 analogue from (**A**) wild-type and (**B**) nmMLCK deficient mice (n = 12). **P < 0.01, ***P < 0.001.

Aortas from wild type or nmMLCK-deficient mice were isolated and exposed to IH for 6 hours. Then, endothelium-dependent relaxation to acetylcholine was assessed. IH reduced the acetylcholine-induced relaxation in aortas from wild type mice compared to aortas exposed to normoxia (Fig. 6A).

Interestingly, IH failed to affect relaxation in response to acetylcholine in vessels taken from nmMLCK-deficient mice (Fig. 6B). These results suggest that the presence of nmMLCK is essential for IH-induced endothelial dysfunction, even though this kinase deletion affect endothelial function independently of IH condition.

## Discussion

We report that the experimental protocol of IH used in the present study reproduces *in vitro* the features of endothelial dysfunction and inflammation observed in OSA, notably the increased of ROS, the activation of p65-NFκB, the release of inflammatory cytokines such as IL-6, the transendothelial migration of monocytes and the endothelial dysfunction on mouse aortas. Thus, *in vitro* IH arises as a valuable model for studying the mechanisms involved in the IH-induced early stages of atherosclerosis as seen in OSA patients. In this context, nmMLCK inhibition, with two inhibitors acting through two different mechanisms, prevents IL-6 secretion downstream of p65-NFκB activation, the increase of transendothelial migration and the endothelial dysfunction in aortas induced by IH. We provide evidence of the new role of nmMLCK with regard to its interaction with VAMP-associated vesicles in the release of IL-6.

The IH protocol used in this study is composed of 6 cycles of intermittent hypoxia with a variation of O_2_ from 5% to 21% every 30 minutes. This model differs from the pathophysiology of OSA patients in terms of hypoxic events. Indeed, in OSA patients, the duration of hypoxic period is about 10 seconds repeated many times during the night, defining the apnea/hypopnea index which measures the number of apneas and hypopneas per hour of sleep. Patients suffering from a severe form of OSA have an apnea/hypopnea index greater than 30 per hour of sleep^16^. Although the model of IH is different of the pathological condition, we have been able to reproduce inflammation characteristics highlighted in OSA. Indeed, vascular endothelial cells harvested from patients exhibit an increase of oxidative stress rate compared to those harvested from control subjects^17^. OSA patients have a high rate of circulatory inflammatory cytokines like IL-6, TNFα or the C-reactive protein^18^ leading to the establishment of a systemic inflammation. The elevated levels of these cytokines were significantly correlated to an increased in intima/media thickness, a marker of atherosclerotic process^18^. Furthermore, the intima/media thickness was positively correlated to the apnea/hypopnea index^19^. The systemic inflammation could then ultimately lead to atherosclerotic damages in OSA patients. In animal models, it has been shown that mice exposed to IH had a higher oxidative stress in several tissues as lungs and liver^20^, and higher activation of NFκB contributing to the atherosclerosis^21^. Moreover, IH increased atherosclerotic lesions in mouse aortas associated with an increase of adhesion markers on vessels leading to infiltration of leukocytes within the vascular wall^7^. Of particular interest in the present study, we have detected an increase of these markers of inflammation following IH exposure, including an increase of NO and ROS production, an activation of the inflammatory pathway p65-NFκB associated with the secretion of IL-6 by endothelial cells, and an increase of *in vitro* transendothelial migration of monocytes. All of these data validate the experimental protocol of IH used here as relevant to study inflammation in regard to OSA. These results also reinforce the effects of IH on endothelial cells, particularly in terms of endothelial inflammatory activation. We also showed that IH increased NO production resulting from an activation of eNOS. Data in the literature concerning the consequences of IH on NO pathway are contradictory depending on the model and the protocol used to induce IH. Indeed, in skin biopsies, it has been reported an increase in eNOS mRNA expression in severely hypoxemic OSA patients^16^. At the vascular level, an increase of eNOS expression and activity has been described in carotid arteries from fetal rabbits exposed to IH^22^. Also, in hypertensive rats, IH induced formation of available NO stores and enhanced the capacity of aortic rings to store NO improving endothelial function^23^. In contrast, in human microvascular dermal endothelial cells, IH exposure induced a decrease in eNOS expression^24^, whereas no effect or a slight increase in eNOS expression was described in human carotid arterial endothelial cells or in aortas from IH-exposed mice, respectively. Finally, Jelic and colleagues^3^ have demonstrated that, in isolated circulating endothelial cells from OSA patients, both eNOS expression as well as phosphorylation at the activator residue was decreased in OSA patients compared with healthy subjects. Nevertheless, an increase in protein nitration in these cells was observed^3^, in accordance with the present study. Indeed, we have observed that *in vitro* IH increased the protein nitration in HAoECs. However, the identity of the nitrated proteins remains to be assessed. Nonetheless, the concomitant increase of both NO and ROS production as detected by DHE staining and the consequent protein nitration suggest that IH induced deleterious effects in endothelial cells. It should be noted that mitochondrial ROS production was not modified by IH exposure as indicated by no changes in MitoSox™ fluorescence (data not shown).

The pharmacological inhibition of nmMLCK prevented some of the IH-induced alterations observed in HAoECs. Firstly, nmMLCK inhibition prevented the IH-induced increase of NO production without affecting the production of ROS or the activation of p65-NFκB. In another model of inflammation, we have already described such a role as nmMLCK-deficiency protects against LPS-induced NO overproduction in aorta^10^. This prevention was associated with decreased expression of iNOS within the vascular wall. In the present study, the overproduction of NO was independent of iNOS expression. nmMLCK preferentially regulated the IH-induced phosphorylation of eNOS at the activator site. Moreover, in a model of LPS, others and we demonstrated that nmMLCK regulated the activation of p65-NFκB^10,11^. LPS binds to the toll-like receptor 4 and activates some associated co-factors such as Myd88, IRAK6, which consequently leads to p65-NFκB stimulation. In this signaling pathway, nmMLCK modulates the active phosphorylation of p65-NFκB. Interestingly, in the present study, nmMLCK did not modify IH-induced activation of p65-NFκB. In contrast to LPS, the molecular mechanisms of IH-induced NFκB activation may not dependent on nmMLCK activity. Some studies report that ROS are upstream the activation of NFκB, inasmuch IH failed to activate this transcription factor after antioxidant treatments^25^.

We also show here that nmMLCK regulates transendothelial migration of monocytes in response to IH. This result is consistent with the literature with regards to the atherosclerotic process in OSA. The transmigration of inflammatory cells is a key event in the pathogenesis of atherosclerosis and mechanisms involved in this phenomenon include an increase of endothelial permeability. Indeed, either thrombin or LPS treatment increased *in vitro* and *in vivo* endothelial permeability^26^. The molecular mechanisms include cytoskeletal rearrangement via the increase of intracellular calcium. The later induces the calcium/calmodulin-dependent activation of nmMLCK in endothelial cells and leads to endothelial contraction. In a model of septic shock, Wainwright and colleagues^26^ have demonstrated that LPS injection increases infiltration of leukocytes in lungs from wild-type mice whereas nmMLCK deficiency protects against this infiltration. The interaction between leukocytes and endothelial cells activates Src, which in turn can phosphorylate the kinase Pyk2 implicated in the disorganization of the adherens junctions^27^. Moreover, Src can phosphorylate nmMLCK at the residues tyrosine 464 and 471 increasing the activity of nmMLCK and the cellular junctions opening^28^. Thus, nmMLCK in endothelial cells is important in the transmigration of leukocytes across endothelial barrier. In a model of mice fed with high-fat diet, pharmacological inhibition of nmMLCK in monocytes protects against the high-fat diet-induced transendothelial migration^12^. Thus, nmMLCK expressed in endothelial cells, but also in leukocytes, might be a potential target to prevent early processes of atherosclerosis observed in OSA.

Of particular importance in this study is the role of nmMLCK on the regulation of IL-6 secretion by endothelial cells during IH. The release of inflammatory cytokines in OSA is well documented and contributes to the pathophysiological mechanisms of the disease. However, to our knowledge, the molecular mechanisms implicated in the release of cytokines in OSA are not well documented. In the present study, ML-7 inhibited the release of IL-6 induced by IH but not p65-NFκB activation. Thus, activation of nmMLCK is mandatory for its release. Indeed, the inhibitor of p65-NFκB, Bay 11-708, abrogated the release of IL-6. One can advance the hypothesis that IH activates the inflammatory NFκB pathway and the later probably interacts downstream either directly with nmMLCK or with other binding partners of nmMLCK to induce the release of IL-6. These partners include Pyk2, Src or other proteins^29,30^. Further studies analyzing the interactome of nmMLCK are needed to characterize the identity of these partners. Nevertheless, the present study is in favor of the hypothesis that p65-NFκB, nmMLCK and nmMLCK with its partner’s axis may be involved in the release of IL-6 induced by IH under the experimental condition used.

A novelty of the mechanism is that IL-6 secretion is associated with a reorganization of VAMP-dependent vesicles. In endothelial cells, inflammatory cytokines can be stored in vesicles to be secreted by cells after inflammatory stimulation. Moreover, in a model of pain induced by an injection of LPS, a role of Rho kinases pathway in the release of TNFα and IL-1β has been reported in the spinal cord of mice^31^. Also, cdc42 and RalA belonging to the Rho GTPase family are implicated in TNFα-induced monocyte chemoattractant protein 1 traffic release in microvascular endothelial cells^32^. Thus, one can advance the hypothesis that nmMLCK participates in cytoskeletal reorganization as the Rho kinases family and could be implicated in intracellular trafficking of vesicles-containing cytokines allowing their release. In the present study, IH leads to the formation of VAMP-associated vesicles with a decrease of IL-6 within endothelial cells through the activity of nmMLCK, since its pharmacological inhibition shuts out VAMP reorganization and prevents IL-6 secretion. nmMLCK can act as a multifunctional protein which interacts with different binding partners. In a previous study, we have demonstrated that nmMLCK physically interacts with p65-NFκB and plays a pivotal role in vascular inflammatory diseases^10^. Other authors have depicted an interaction of nmMLCK with cytoskeletal proteins as cortactin, but also with the regulatory p60Src, leading to the assembly and activation of the NADPH oxidase^29^. The results of the present study reinforce the idea that nmMLCK could be a platform interacting with binding partners, modulating their activities and/or spatial reorganization and therefore could be an interesting target to prevent OSA-related inflammatory environment associated with cytokines release.

Endothelial dysfunction is also a critical consequence of IH. Indeed, an impairment of vascular relaxation has already been described in OSA patients illustrated by a decrease in flow-mediated dilation in OSA patients compared to healthy subjects^33^. A decrease of the migration of endothelial cells has also been established when cells were stimulated with OSA patients’ serum^34^. Our group has also established that injection of mice with circulating microparticles from OSA patients decrease vascular relaxation of aortas in response to acetylcholine whereas microparticles from healthy controls have no effects^35^. Moreover, flow-mediated dilation of small mesenteric arteries was altered after treatment with microparticles from patients. In the present study, *in vitro* exposure of aortas from wild type to IH impaired vascular relaxation. Interestingly, nmMLCK deficiency protects against IH-induced endothelial dysfunction. In contrast to our former studies, LPS induced endothelial dysfunction in wild type and nmMLCK-deficient mice^10^. These discrepancies could be explained by the fact that, in the present study, we observed an increase of *in vitro* NO production and eNOS activity in endothelial cells. Several differences are noteworthy. First, on endothelial cells, we only studied the effect of pharmacological inhibition of nmMLCK and not the deletion of the kinase. It is of importance because previous study already established a major role of the presence of nmMLCK instead of its activity^12^. Second, relaxation experiments were made within aortas in which smooth muscle cells, and/or fibroblasts could be affected by IH and therefore might explain the observed differences. Nevertheless, we observed that nmMLCK is involved in IL-6 release which is known to induce endothelial dysfunction. Thus, inhibition of IL-6 release might participate in the protection of endothelial function by nmMLCK in the model of IH.

In summary, we provide evidence that, in an experimental model of OSA, nmMLCK is a potential molecular target to counteract early stages of atherosclerosis, such as endothelial dysfunction, inflammation through its capacity to increase transendothelial migration of monocytes and, most importantly, through the reorganization of VAMP-dependent vesicles associated with the release of IL-6. The present study underscores a novel role for nmMLCK in inflammatory activation. These findings could be essential in the design of new therapeutic strategies targeting nmMLCK to fight against inflammation in the pathophysiological process of OSA.

## Methods

### Cell culture and IH exposure

Human aortic endothelial cells (HAoECs) were grown in endothelial basal medium supplemented with 10% of fetal bovine serum and 1% of antibiotics. After the confluence, cells were exposed to an experimental protocol of IH already used in the literature^35,36^. Briefly, the IH protocol is composed of 6 cycles of 30 minutes of hypoxia with 5% of O_2_ followed by 30 minutes of normoxia with 21% of O_2_. For normoxia, HAoECs were exposed to 21% of O_2_ during 360 minutes. In some experiments, the pharmacological inhibitor of nmMLCK, ML-7 (Sigma-Aldrich, St Quentin Fallavier, France), were added at a maximally active concentration (5 μM), 30 minutes before the IH protocol^12^. In order to confirm the effect of ML-7, some experiments were performed using the MLCK peptide inhibitor 18 (PIK, Calbiochem, La Jolla, CA) added at a maximally active concentration (150 µM). PIK is a membrane permeant inhibitor composed of 9 amino acids, binding to the catalytic domain of nmMLCK and, thus, inactivating the enzyme^37^.

### Nitric oxide (NO) production by HAoECs by electronic paramagnetic resonance

Detection of NO production by HAoECs was performed using a previously described electronic paramagnetic resonance technique with ferrous diethyldithiocarbamate (DETC, Sigma-Aldrich) as a spin trap^38^. Briefly, cells were grown until confluence in 6-wells plate and exposed in normoxia or IH. After the stimulation, 100 µM of Fe(DETC)_2_ were added to the cells and incubated 45 minutes at 37 °C. Cells were scratched and were then frozen and conserved at −80 °C. Analyses were performed on a tabletop x-band spectrometer Miniscope (Magnettech, Berlin, Germany).

### Evaluation of reactive oxygen species (ROS) production by HAoECs by flow cytometry

HAoECs were seeded into 12-wells plate and grown until confluence. Then, HAoECs were exposed to normoxia or IH for 6 hours, in presence or in absence of ML-7 (5 μM). After the stimulation, cells were washed once with PBS and incubated with dihydroethidium (3 μM, Sigma-Aldrich) for 30 minutes at 37 °C. Cells were detached with trypsin and suspended into 200 μl of PBS. Fluorescence was then determined by flow cytometry 500 MPL System (Beckman Coulter, Villepinte, France) with the MXP software (Beckman Coulter).

### Western Blotting

After IH exposure, HAoECs were lysed to extract protein contents. 40 μg of protein was separated by 4–12% gel electrophoresis (Invitrogen, Carlsbad, CA). Then, proteins were transferred to nitrocellulose membranes and probed with rabbit polyclonal antibody for endothelial NOS (eNOS, 1:1000, BD Pharmingen, San Jose, CA), phosphorylated (serine 1177) eNOS (1:1000, Cell Signaling, Danvers, MA), subunit p65 of NFκB (1:1000, Abcam, Cambridge, United Kingdom), phosphorylated (serine 536) p65-NFκB (1:1000, Santa Cruz Biotechnology, Dallas, TX), inhibitor of κBα (IκBα; 1:1000, US Biological, Salem, MA) and phosphorylated (serine 32) IκBα (1:1000, US Biological). After washing, bound antibodies were detected with a secondary peroxidase-conjugated anti-rabbit antibody (1:20 000, US Biological) or anti-mouse antibody (1:5000, US Biological). The same membrane was used to determine β-actin expression using a polyclonal antibody (1:5000, Sigma-Aldrich). The bands were visualized using the enhanced chemiluminescence system and quantified by densitometry. Unprocessed images of all immunoblots are in Supplementary Information.

### Quantitative real-time reverse transcription–polymerase chain reaction analysis

After IH exposure, HAoECs were used to isolate mRNA of inflammatory markers in order to evaluate their expression. The analyze of RT-PCR were carried out by *Plateforme d’Analyse Cellulaire et Moléculaire* in Angers University using a Chromo 4TM (Bio-Rad, Hercules, CA) and SYBR Green detection.

### Enzyme-linked immunosorbent assay (ELISA)

After IH exposure in absence or presence of ML-7 (5 μM), we harvested supernatant of HAoECs. We measured concentration of IL-6 using ELISA kit (Raybiotech, Norcross, GA) according to the manufacturer protocol.

### Staining and imaging with confocal microscopy

After exposure of HAoECs to one cycle of IH, cells were incubated (1 hour, room temperature) in blocking buffer (1% non-fat dry milk in physiological buffer solution). After 3 washes, cells were incubated overnight (4 °C) with anti-IL-6 and anti-VAMP (1:100, Santa Cruz Biotechnology) antibodies. Three washes were followed by incubation (2 hours, room temperature) with secondary mouse fluorescence-labelled antibody Fluoprobes FP 546 and secondary rabbit fluorescence-labelled antibody Fluoprobes 488 (1:500, Interchim, San Diego, CA). Images were then taken using confocal microscope and analyzed with Zeiss software.

### Myography

To evaluate the effects of IH on endothelial function, we isolated aortas from wildtype or nmMLCK-deficient mice^26^. Aortas were exposed to IH as described above. Then, mouse aortas were mounted on a wire myograph (Aarhus, Denmark). Mechanical activity was recorded isometrically using a force transducer. The experiments were performed at 37 °C with physiological saline of the following millimolar composition (NaCl 130, NaHCO_3_ 14.9, KCl 3.7, KH_2_PO_4_ 1.2, MgSO_4_·7H_2_O 1.2, CaCl_2_·H_2_O 1.6, glucose 11) continuously bubbled with 95% O_2_ and 5% CO_2_. The functionality of the endothelium was assessed by the ability of acetylcholine (Sigma-Aldrich) to induce relaxation. Then, cumulative addition of acetylcholine was assessed on vessel pre-contracted with the thromboxane analogue (U-46619, Sigma-Aldrich) at 80% of its maximal contraction. All animal care and treatment procedures were performed in accordance with institutional guidelines. Protocols were approved by the French Animal Care Committee in accordance with European regulations (Comité d’Ethique pour l’Expérimentation Animale; Pays de la Loire 2012.94).

### Adhesion and Transmigration assay

For adhesion and transmigration assays, co-culture of HAoECs and the monocytic cell line THP-1 was performed. THP-1 were cultured in RPMI 1640 medium supplemented with 10% of fetal bovine serum and 1% of antibiotics. Then, HAoECs were grown in 6-well plates until 100% confluency. At this point, HAoECs were exposed to IH for 6 hours in absence or in presence of ML-7 (5 µM). At the end of the IH exposure, THP-1 monocytes at a density of 3 × 10^5^ cells/ml were added to each well. Adhesion was allowed for 1 hour before several washes and fixation with 4% paraformaldehyde. Photos were taken, and the number of THP-1 cells per field was count for each condition.

Transmigration assay were performed using Transwell (8 µm pore size, Corning, Corning, NY). HAoECs were seeded in upper chamber at density of 2 × 10^5^ cells/ml and were grown until confluence. The cells were exposed to IH for 6 hours in absence or presence of ML-7 (5 µM). At the end of the stimulation, 3 × 10^5^ THP-1 were added to the upper chamber of the Transwell and were allowed to transmigrate for 24 hours. Then, medium of the lower chamber were harvested, centrifuged and the cells were counted with Trypan Blue.

### Statistical analysis

Results are expressed as means ± S.E.M. For myography experiments, n represents the number of mice. Vascular reactivity was compared using a two-way ANOVA with repeated measurements. Unpaired Student’s t-tests were used for Western blots. The levels of NO were compared using one-way ANOVA followed by a Newman–Keuls multiple comparison post-hoc test. P < 0.05 was considered to be significant.

## Electronic supplementary material

Supplementary information

## References

[1] Gonzaga C, Bertolami A, Bertolami M, Amodeo C, Calhoun D (2015). Obstructive sleep apnea, hypertension and cardiovascular diseases. J. Hum. Hypertens..

[2] Badran, M., Ayas, N. & Laher, I. Cardiovascular complications of sleep apnea: role of oxidative stress. *Oxid. Med. Cell. Longev*. 985258 (2014). 10.1155/2014/985258 PMC3964889 24734153

[3] Jelic S (2010). Vascular inflammation in obesity and sleep apnea. Circulation.

[4] Ryan S, McNicholas WT, Taylor CT (2007). A critical role for p38 map kinase in NF-kappaB signaling during intermittent hypoxia/reoxygenation. Biochem. Biophys. Res. Commun..

[5] Ryan S, Taylor CT, McNicholas WT (2005). Selective activation of inflammatory pathways by intermittent hypoxia in obstructive sleep apnea syndrome. Circulation.

[6] Chuang LP, Chen NH, Lin Y, Ko WS, Pang JH (2016). Increased MCP-1 gene expression in monocytes of severe OSA patients and under intermittent hypoxia. Sleep Breath..

[7] Arnaud C (2011). The inflammatory preatherosclerotic remodeling induced by intermittent hypoxia is attenuated by RANTES/CCL5 inhibition. Am. J. Respir. Crit. Care Med..

[8] Goeckeler ZM, Wysolmerski RB (1995). Myosin light chain kinase-regulated endothelial cell contraction: the relationship between isometric tension, actin polymerization, and myosin phosphorylation. J. Cell Biol..

[9] Ralay Ranaivo H (2007). Protection against endotoxic shock as a consequence of reduced nitrosative stress in MLCK210-null mice. Am. J. Pathol..

[10] Recoquillon S (2015). Interaction in endothelium of non-muscular myosin light chain kinase and the NF-κB pathway is critical to lipopolysaccharide-induced vascular hyporeactivity. Clin. Sci..

[11] Tauseef M (2012). TLR4 activation of TRPC6-dependent calcium signaling mediates endotoxin-induced lung vascular permeability and inflammation. J. Exp. Med..

[12] Sun C, Wu MH, Yuan SY (2012). Nonmuscle myosin light-chain kinase deficiency attenuates atherosclerosis in apolipoprotein E-deficient mice via reduced endothelial barrier dysfunction and monocyte migration. Circulation.

[13] Karin M, Ben-Neriah Y (2000). Phosphorylation meets ubiquitination: the control of NF-kappaB activity. Annu. Rev. Immunol..

[14] Ryan S, Taylor CT, McNicholas WT (2009). Systemic inflammation: a key factor in the pathogenesis of cardiovascular complications in obstructive sleep apnoea syndrome?. Postgrad. Med. J..

[15] Lee KS (2014). Functional role of NF-κB in expression of human endothelial nitric oxide synthase. Biochem. Biophys. Res. Commun..

[16] Kaczmarek E (2013). Molecular biomarkers of vascular dysfunction in obstructive sleep apnea. PLoS One.

[17] Jelic S (2008). Inflammation, Oxidative Stress, and Repair Capacity of the Vascular endothelium in Obstructive Sleep Apnea. Circulation.

[18] Ciccone MM (2014). Correlation between inflammatory markers of atherosclerosis and carotid intima-media thickness in Obstructive Sleep Apnea. Molecules.

[19] Drager LF (2005). Early Signs of Atherosclerosis in Obstructive Sleep Apnea. Am. J. Respir. Crit. Care Med..

[20] da Rosa, D.P. *et al*. Simulating sleep apnea by exposure to intermittent hypoxia induces inflammation in the lung and liver. *Mediators Inflamm*. 879419 (2012). 10.1155/2012/879419 PMC3513737 23226929

[21] Song D (2012). Chronic intermittent hypoxia induces atherosclerosis by NF-κB-dependent mechanisms. Biochim. Biophys. Acta..

[22] Chen YF, Wang ZH, Chen ZK, Lv GR, Ferrari M (2013). Intermittent maternal hypoxia has an influence on regional expression of endothelial nitric oxide synthase in fetal arteries of rabbits. Pediatr. Res..

[23] Manukhina EB, Jasti D, Vanin AF, Downey HF (2011). Intermittent hypoxia conditioning prevents endothelial dysfunction and improves nitric oxide storage in spontaneously hypertensive rats. Exp. Biol. Med..

[24] Zhong A, Xiong X, Shi M, Xu H (2016). Roles of interleukin (IL)-6 gene polymorphisms, serum IL-6 levels, and treatment in obstructive sleep apnea: a meta-analysis. Sleep Breath..

[25] Zhang J, Zheng L, Cao J, Chen B, Jin D (2015). Inflammation induced by increased frequency of intermittent hypoxia is attenuated by tempol administration. Braz. J. Med. Biol. Res..

[26] Wainwright MS (2003). Protein kinase involved in lung injury susceptibility: evidence from enzyme isoform genetic knockout and *in vivo* inhibitor treatment. Proc. Natl. Acad. Sci..

[27] Allingham MJ, van Buul JD, Burridge K (2007). ICAM-1-mediated, Src- and Pyk2-dependent vascular endothelial cadherin tyrosine phosphorylation is required for leukocyte transendothelial migration. J. Immunol..

[28] Birukov KG (2001). Differential regulation of alternatively spliced endothelial cell myosin light chain kinase isoforms byp60(Src). J. Biol. Chem..

[29] Usatyuk PV (2012). Novel role for non-muscle myosin light chain kinase (MLCK) in hyperoxia-induced recruitment of cytoskeletal proteins, NADPH oxidase activation, and reactive oxygen species generation in lung endothelium. J. Biol. Chem..

[30] Wadgaonkar R (2005). Intracellular interaction of myosin light chain kinase with macrophage migration inhibition factor (MIF) in endothelium. J. Cell. Biochem..

[31] Wang, C. *et al*. Inhibition of the Rho/Rho kinase pathway prevents lipopolysaccharide-induced hyperalgesia and the release of TNF-α and IL-1β in the mouse spinal cord. *Sci. Rep*. 14553 (2015). 10.1038/srep14553 PMC4586490 26416580

[32] Langert, K.A., Pervan, C.L. & Stubbs, E.B. Jr. Novel role of Cdc42 and RalA GTPases in TNF-α mediated secretion of CCL2. *Small GTPases* e29260 (2014). 10.4161/sgtp.29260 PMC4205150 24911990

[33] Tanriverdi H (2006). Aortic stiffness, flow-mediated dilatation and carotid intima-media thickness in obstructive sleep apnea: non-invasive indicators of atherosclerosis. Respiration.

[34] Hoffmann M (2017). Serum of obstructive sleep apnea patients impairs human coronary endothelial cell migration. Arch. Med. Sci..

[35] Priou P (2010). Endothelial dysfunction and circulating microparticles from patients with obstructive sleep apnea. Am. J. Pathol..

[36] Emin, M. *et al*. Increased internalization of complement inhibitor CD59 may contribute to endothelial inflammation in obstructive sleep apnea. *Sci. Transl. Med*. 8 (2016). 10.1126/scitranslmed.aad0634 PMC5485919 26738794

[37] Zolotarevsky Y (2002). A membrane-permeant peptide that inhibits MLC kinase restores barrier function in *in vitro* models of intestinal disease. Gastroenterology.

[38] Stoclet J (1999). Induction of nitric oxide synthase and dual effects of nitric oxide and cyclooxygenase products in regulation of arterial contraction in human septic shock. Circulation.

