# Supplementary material for: Non-muscular myosin light chain kinase triggers intermittent hypoxia-induced interleukin-6 release, endothelial dysfunction and permeability
Source: Sci Rep. 2017 Oct 20;7:13664. doi: 10.1038/s41598-017-13268-5 (PMC5651916; doi:10.1038/s41598-017-13268-5)
Supplement: Supplementary file 1 — Supplementary information [file 41598_2017_13268_MOESM1_ESM.pdf]

Non-muscular myosin light chain kinase triggers intermittent hypoxia-induced interleukin-6 release, endothelial dysfunction and permeability

Sylvain Recoquillon, Manuel Gómez-Guzmán, Marion Rodier, Camille Koffi, Mathieu Nitiéma, Frédéric Gagnadoux, M. Carmen Martínez, Ramaroson Andriantsitohaina

p-eNOS (6 hours) in figure 2

1 = Ctl
2 = ML-7
3 = IH
4 = IH + ML-7

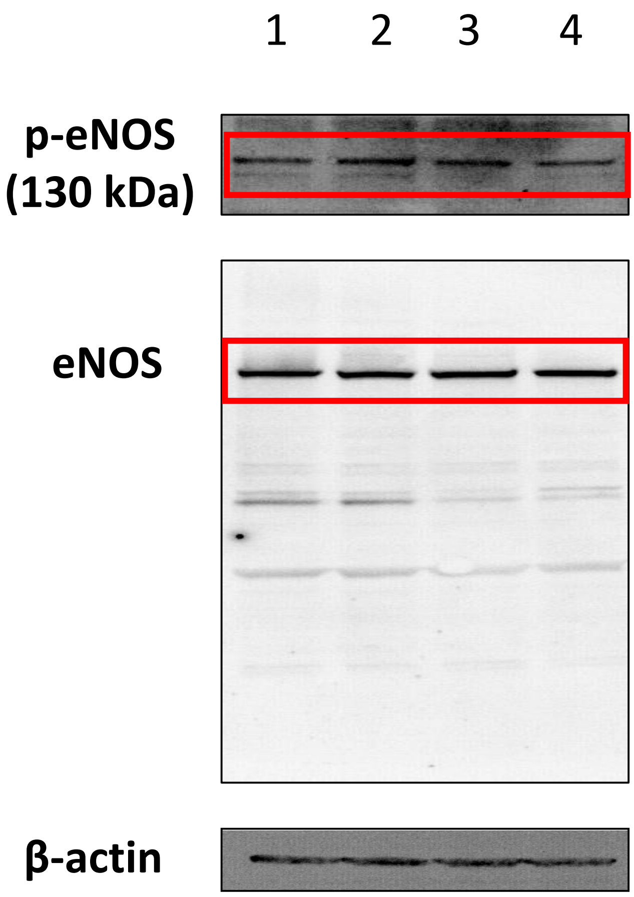

Supplementary Figure 1. Unprocessed images of the key immunoblots of Figure 2. Boxes indicate image areas shown in the indicated panels.

IκB (6 hours) in figure 3

1 = Ctl
2 = ML-7
3 = IH
4 = IH + ML-7

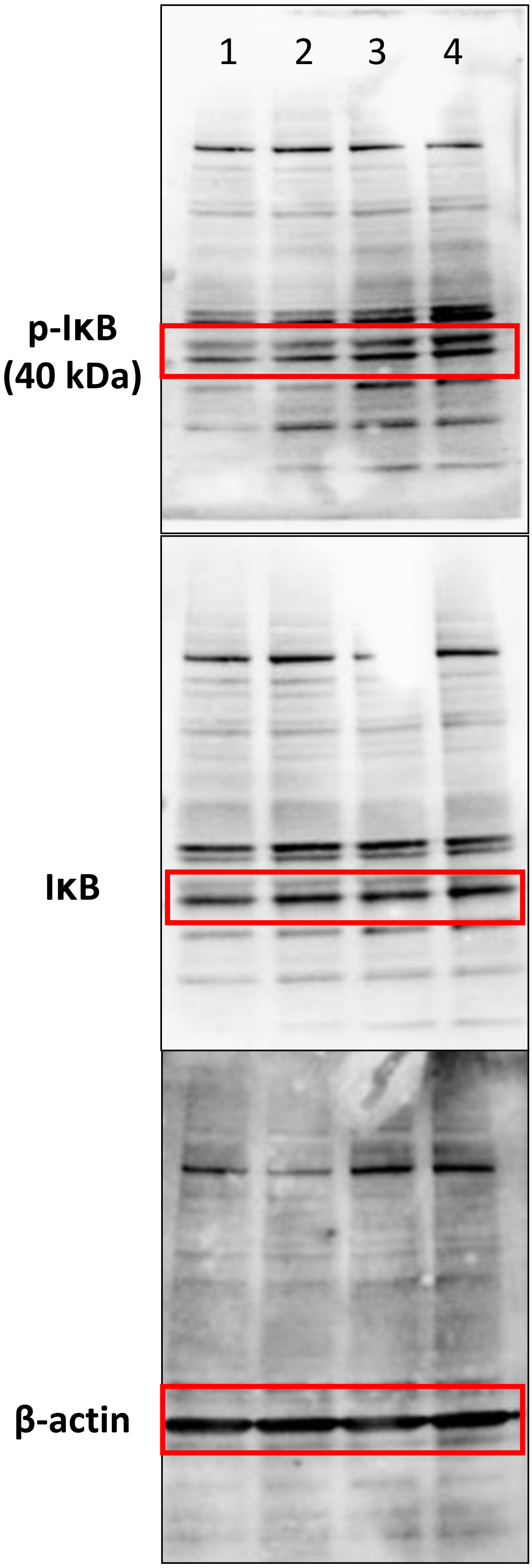

Supplementary Figure 2. Unprocessed images of the key immunoblots of Figure 3. Boxes indicate image areas shown in the indicated panels.

p65 (6 hours) in figure 3

1 = Ctl
2 = ML-7
3 = IH
4 = IH + ML-7

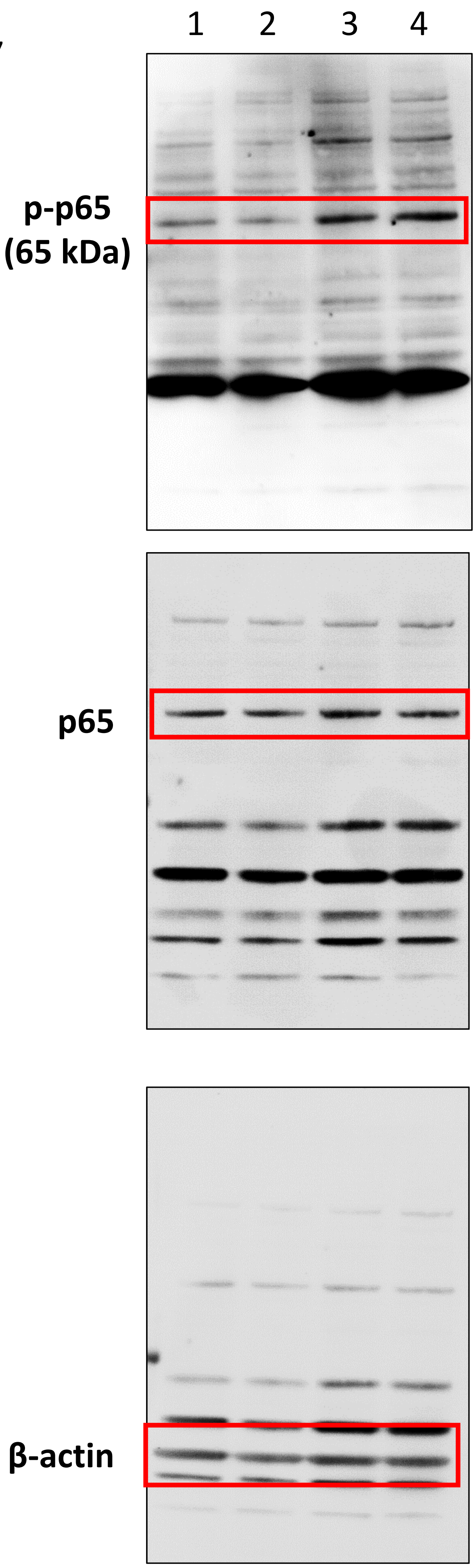

Supplementary Figure 3. Unprocessed images of the key immunoblots of Figure 3. Boxes indicate image areas shown in the indicated panels.

p-p65 (6 hours) in figure 4

1 = Ctl
2 = BFA
3 = IH
4 = IH + BFA

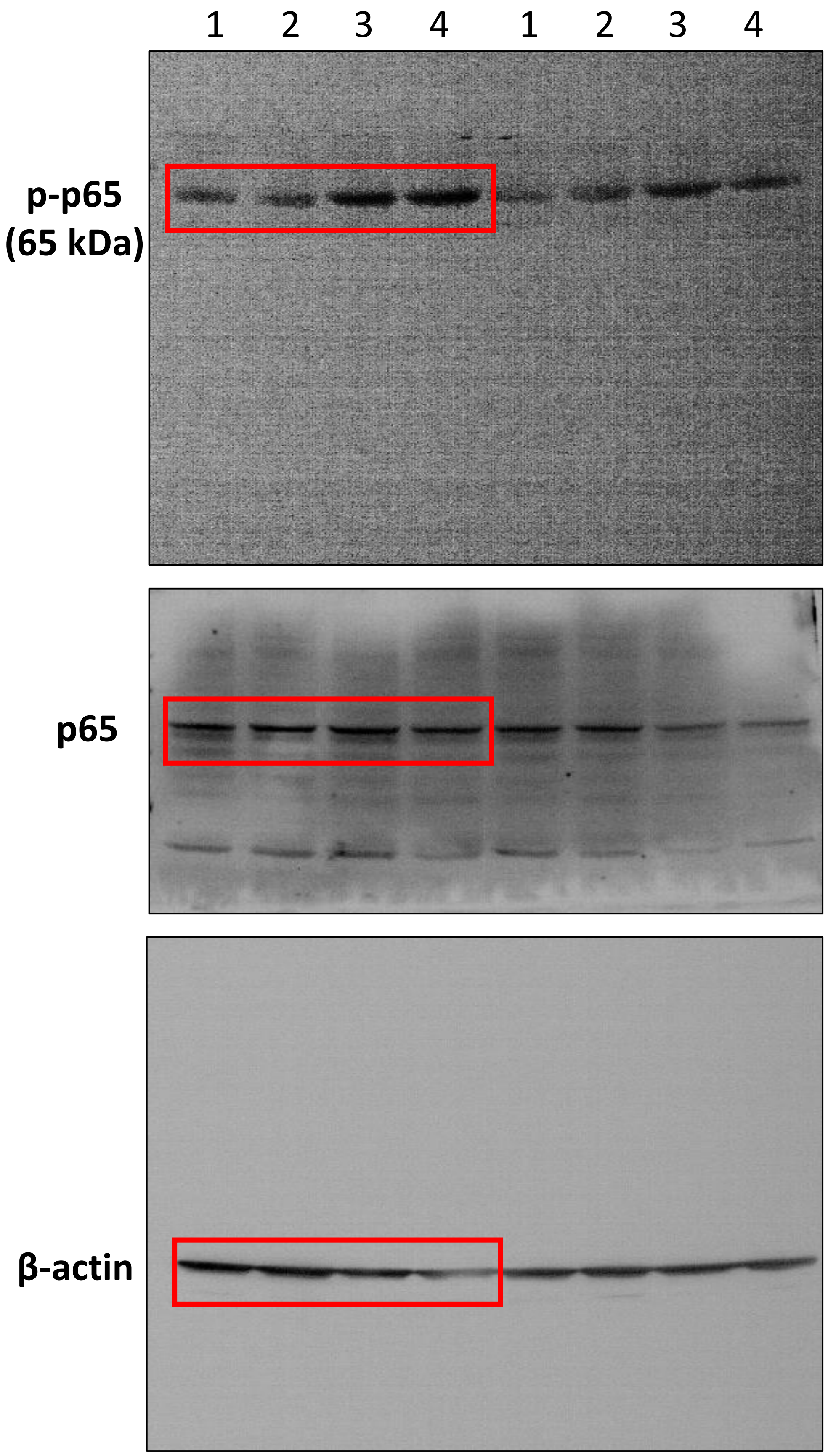

Supplementary Figure 4. Unprocessed images of the key immunoblots of Figure 4. Boxes indicate image areas shown in the indicated panels.
